# Sleep disturbance and its associations with night shift work, anxiety symptoms, and depressive symptoms among urban operation-supporting workers: a cross-sectional study

**DOI:** 10.3389/fpubh.2026.1935598

**Published:** 2026-09-29

**Authors:** Yilin Quan, Jingwen Yang, Fengqiong Chen, Zhiqi Wang, Anchao Song, Mengliang Ye

**Affiliations:** 1 Department of Epidemiology and Health Statistics, School of Public Health, Chongqing Medical University, Chongqing, China; 2 Chongqing Municipal Center for Disease Control and Prevention, Chongqing, China; 3 Department of Neurosurgery, The First Affiliated Hospital of Chongqing Medical University, Chongqing, China

**Keywords:** environmental sanitation workers, healthcare workers, mental health, night shift work, occupational health, sleep disturbance, transportation workers

## Abstract

**Background:**

Sleep disturbance is common among occupational populations and may vary by work schedules and psychological burdens. However, evidence among urban operation-supporting occupational groups remains limited. This study aimed to compare the prevalence of sleep disturbance among healthcare, environmental sanitation, and transportation workers in Chongqing, China, and to examine the associations of night shift work, anxiety, and depressive symptoms with sleep disturbance.

**Methods:**

This study was based on the *Health Literacy Surveillance Project for Key Occupational Groups in Chongqing* (June–September 2022). Overall, 3,667 front-line workers were included. Sleep disturbance was assessed using the *Chinese Center for Disease Control and Prevention sleep quality scale*. Anxiety and depressive symptoms were assessed using the *Generalized Anxiety Disorder-7 scale* and the *Patient Health Questionnaire-9*, respectively. Modified Poisson regression models with robust variance estimation were used to estimate adjusted prevalence ratios (aPRs) and 95% confidence intervals (CIs). Interaction terms were further included to examine occupational group differences.

**Results:**

The prevalence of sleep disturbance was 94.1, 85.1, and 92.8% among healthcare, environmental sanitation, and transportation workers, respectively. After adjustment, environmental sanitation workers had a lower prevalence of sleep disturbance than healthcare workers (aPR = 0.904, 95% CI: 0.862–0.947, *p* < 0.001), whereas transportation workers showed no significant difference (aPR = 0.996, 95% CI: 0.956–1.037, *p* = 0.839). Night shift work (aPR = 1.025, 95% CI: 1.003–1.048, *p* = 0.028) and anxiety symptoms (aPR = 1.094, 95% CI: 1.073–1.116, *p* < 0.001) were associated with a higher prevalence of sleep disturbance. Depressive symptoms showed no statistical significance in the fully adjusted main model (aPR = 1.018, 95% CI: 0.999–1.038, *p* = 0.068). The environmental sanitation sector significantly interacted with anxiety symptoms (aPR = 1.082, 95% CI: 1.030–1.136, *p* = 0.002), suggesting a stronger anxiety-sleep disturbance association. No significant interactions were observed between occupational group and depressive symptoms or night shift work.

**Conclusion:**

Sleep disturbance was prevalent among urban operation-supporting occupational groups. Night shift work and anxiety symptoms were associated with sleep disturbance, with anxiety showing the most stable association. Depressive symptoms showed a weaker, model-dependent association. Sleep and mental health interventions may need to consider occupational context.

## 1 Introduction

Sleep disturbance is one of the common public health problems worldwide and has been shown to be closely associated with various adverse health outcomes, including cardiovascular disease, metabolic abnormalities, and psychological disorders (1). Previous studies have shown that sleep problems are particularly prominent among occupational populations, especially in industries that require continuous service provision (2, 3), such as healthcare, transportation, and other urban operation-supporting occupations, defined as front-line jobs essential for maintaining continuous urban public services. The work patterns of these occupations often involve night or rotating shifts. Night or rotating shift work may increase the risk of sleep disturbance through circadian disruption (4–7). Previous cohort and review studies have consistently reported an association between night or rotating shift work and sleep disturbance (8, 9). Night shifts may also cause marked sleepiness and increase the risk of safety incidents (10).

Work environments and job characteristics vary across occupations and may be differentially associated with sleep health. Taking the healthcare industry as an example, workers often face high work intensity, emotional burden, and frequent night shifts. Studies have shown that their risk of sleep disturbance is increased (11), while long working schedules, such as 12-h shifts, may further aggravate sleep deprivation and fatigue (12). Transportation workers may work for prolonged periods while maintaining a high level of alertness, and sleep deprivation and sleepiness may increase the risk of traffic accidents (3, 13, 14). In contrast, environmental sanitation workers are mainly engaged in outdoor physical labor and may have early or irregular work schedules, which may also affect sleep (6). High occupational physical activity may promote sleep onset; however, it may also contribute to sleep fragmentation or non-restorative sleep due to accumulated fatigue (15). In addition, outdoor sanitation work may involve specific environmental discomfort and irregular working schedules, which may also affect sleep (16). Several population-based studies have also found differences in sleep duration, sleep quality, and related problems across occupational categories. However, findings on differences in sleep disturbance across occupations remain inconsistent (17, 18), suggesting the need for further comparison within a unified research framework.

Specifically, anxiety and depressive symptoms are closely associated with sleep disturbance, and previous systematic reviews have suggested bidirectional relationships among these conditions (19–21). Previous studies have consistently reported positive associations between depression and sleep disturbance (22, 23); anxiety symptoms have also been confirmed to be significantly associated with sleep disturbance (24). High occupational workload, shift schedules, and work stress may disrupt sleep and may also exacerbate sleep problems through psychological factors such as anxiety and depression. In addition, studies have suggested that multiple negative psychological states may adversely affect sleep health (25). These findings suggest that psychological factors should also be considered when investigating sleep disturbance among occupational populations (21).

Previous studies have mostly focused on single occupational populations or the effects of specific occupational exposure factors on sleep (26, 27), whereas comparative analyses of multiple occupational groups within a unified research framework remain limited (17, 18). Few studies have examined whether the associations of night shift work and psychological factors with sleep disturbance differ across occupational groups. Meanwhile, the combined roles and relative contributions of occupational and psychological factors to sleep disturbance across different occupational contexts have not been systematically evaluated, making it difficult to support industry-specific precision interventions for sleep health.

Therefore, this study included three urban operation-supporting front-line occupational groups in Chongqing, China, namely healthcare, transportation, and environmental sanitation workers. Within a unified survey framework, this study compared the prevalence of sleep disturbance across occupational groups, analyzed the associations of night shift work, anxiety symptoms, and depressive symptoms with sleep disturbance, and further explored whether occupational group modified these associations, aiming to provide evidence for sleep health management and targeted occupational health interventions among different occupational populations.

## 2 Materials and methods

### 2.1 Participants

From June to September 2022, data were collected using a two-stage sampling method. In the first stage, four counties/districts in Chongqing Municipality—Banan, Rongchang, Jiulongpo, and Xiushan—were randomly selected by the National Institute for Occupational Health and Poison Control (NIOHP) of Chinese Center for Disease Control and Prevention (China CDC), using probability proportional to size (PPS) sampling. In the second stage, cluster sampling was used within each selected county/district to recruit front-line workers engaged in three types of urban operation-supporting occupations, including healthcare, environmental sanitation, and transportation. The data were derived from the *Health Literacy Surveillance Project for Key Occupational Groups in Chongqing*. The inclusion criteria were as follows: (1) no history of mental illness or use of psychotropic medication; (2) voluntary signing of informed consent; (3) being a front-line worker; and (4) having worked continuously for at least 1 year. A total of 4,240 workers from the three target occupational groups completed the monitoring questionnaire, including 1,898 healthcare workers, 1,203 environmental sanitation workers, and 1,139 transportation workers. Among them, 573 participants were excluded because they had worked in their current occupation for less than 1 year and therefore did not meet the eligibility criterion for occupational tenure. Finally, 3,667 participants were included in the analysis, including 1,712 healthcare workers, 1,022 environmental sanitation workers, and 933 transportation workers. No missing values were present in the variables included in the main analyses. The final analytic sample accounted for 86.49% of the completed questionnaires from the three target occupational groups.

This study was approved by the Ethics Committee of Chongqing Center for Disease Control and Prevention (Approval No. KY-2024-004-1).

### 2.2 Survey methods

*Basic information questionnaire*. Survey interviews were conducted using the *Individual Questionnaire of the National Occupational Health Literacy Surveillance Survey for Key Groups*. The questionnaire mainly included respondents’ basic demographic information and occupation-related information. Basic demographic information included age, sex, marital status, educational level, and average monthly income. Occupation-related information included occupational category, weekly working hours, and night shift status. Night shift work was assessed using a self-reported binary item. The original monitoring questionnaire did not collect information on night-shift frequency, duration, rotation pattern, cumulative exposure, or recovery time after night shifts.

*Anxiety symptoms*. Anxiety symptoms were assessed using the *Generalized Anxiety Disorder-7 scale* (GAD-7) (28). The scale consists of seven items, and each item is rated by respondents on a four-point scale ranging from 0 to 3. Respondents with a total score of ≥5 were defined as having anxiety symptoms, with higher scores indicating more severe anxiety symptoms. The Cronbach’s alpha coefficient of the scale was 0.913.

*Depressive symptoms*. Depressive symptoms were measured using the *Patient Health Questionnaire-9* (PHQ-9) (29). The questionnaire consists of nine items, with each item rated on a four-point Likert scale ranging from 0 to 3. A total score of ≥10 was defined as depression, with higher total scores indicating more severe depressive symptoms. The Cronbach’s alpha coefficient of the scale was 0.883.

*Sleep disturbance*. Sleep disturbance was used as the outcome variable in this study. Sleep status was assessed using the sleep-quality section of the questionnaire used in the *National Occupational Health Literacy Surveillance Survey for Key Groups*, developed by the Chinese Center for Disease Control and Prevention (30), which evaluated respondents’ actual sleep status over the past 12 months. The section includes three items assessing sleep onset latency, sleep maintenance, and early morning awakening. According to the project scoring protocol, each item is assigned a score of 0 or 1 according to respondents’ answers, with a total score ranging from 0 to 3. Higher scores indicated poorer sleep quality. Respondents with a score of ≥1 were defined as having sleep disturbance, indicating the presence of at least one sleep-related symptom rather than a clinical diagnosis of insomnia or sleep disorder. The Cronbach’s alpha coefficient of the scale was 0.698, suggesting moderate internal consistency. All participants included in the analysis had complete data for the sleep-related items. Given the relatively sensitive threshold of the primary definition and the high prevalence observed, a stricter cut-off of ≥2 was additionally used in sensitivity analyses.

### 2.3 Quality control

Before the formal survey, all investigators received standardized training to ensure that they could proficiently use the survey information system and that their survey skills were standardized and consistent. All respondents from the three occupational groups included in this study signed informed consent forms. The returned questionnaires were strictly screened, and invalid questionnaires with incomplete information, logical inconsistencies, or patterned responses were excluded. The reliability and validity of the questionnaire were assessed according to internationally accepted practices. The database was established using EpiData3.1, and double data entry was performed to ensure data accuracy.

### 2.4 Statistical analysis

Statistical analyses were performed using the SPSSAU online statistical analysis platform (access period: April 27, 2026 to August 24, 2026), and Python was used for result visualization. Continuous variables were first tested for normality. Non-normally distributed continuous variables were described as median and interquartile range [M (P25, P75)], and between-group comparisons were performed using the *Kruskal–Wallis H* test. Categorical variables were presented as numbers and percentages [*n* (%)], and between-group comparisons were performed using the chi-square test. Spearman’s rank correlation analysis was used to assess correlations among major study variables.

Based on previous studies of occupational sleep health (3, 4, 11), the available variables in the monitoring questionnaire, and our conceptual understanding of potential confounding, age, sex, marital status, educational level, monthly income, weekly working hours, and night shift work were selected as covariates in the main models. These variables may be related to occupational group, psychological symptoms, work schedules, and sleep disturbance. Occupational group was treated as the main grouping variable and as a potential effect modifier in the interaction analyses. Self-rated health and sickness absence were not included as primary covariates because their temporal relationship with sleep disturbance was uncertain and they may reflect downstream health status rather than baseline confounding factors.

Because sleep disturbance was highly prevalent, modified Poisson regression models with a log link and robust variance estimation were used to estimate adjusted prevalence ratios (aPRs) and 95% confidence intervals (95% CIs), and to analyze the associations of occupational group, night shift work, anxiety symptoms, and depressive symptoms with sleep disturbance. Separate psychological factor models and a mutually adjusted model were constructed to evaluate the associations between psychological factors and sleep disturbance and the stability of these associations. Interaction terms between occupational group and night shift work, anxiety symptoms, and depressive symptoms were further constructed to examine the effect-modifying role of occupational group. Healthcare workers were used as the reference occupational group because they represented the largest occupational group in the analytic sample and had the highest prevalence of sleep disturbance under the primary definition, providing a meaningful benchmark for comparison with environmental sanitation and transportation workers.

Sensitivity analyses were conducted using a stricter sleep disturbance definition of a total score ≥2. Multicollinearity among independent variables was assessed using variance inflation factors (VIFs). All tests were two-sided, and *p* < 0.05 was considered statistically significant.

## 3 Results

### 3.1 Basic characteristics

A total of 3,667 participants were included in this study and divided into three occupational groups: the healthcare group (*n* = 1,712), environmental sanitation group (*n* = 1,022), and transportation group (*n* = 933). Significant differences were observed among the three groups in demographic characteristics (sex, age, marital status, educational level, and monthly income) and occupation-related characteristics (night shift status and weekly working hours) (all *p* < 0.001).

Overall, women accounted for the majority of the participants (63.3%), but the sex distribution differed markedly across occupational groups. The healthcare and environmental sanitation groups were predominantly female, with the highest proportion of women observed in the healthcare group (81.1%), whereas the transportation group was predominantly male (81.5%). The median age of the total sample was 41 years. Participants in the environmental sanitation group were the oldest, with a median age of 49 years, followed by those in the transportation group (43 years) and the healthcare group (34 years). Most participants were married (81.8%), although the proportion of married participants was relatively lower in the healthcare group (75.6%). The healthcare group had the highest educational level, with 66.5% having a bachelor’s degree or above, whereas the environmental sanitation and transportation groups were mainly composed of participants with a high school/s specialized school or below, accounting for 90.9 and 81.6%, respectively. The proportion of participants with lower monthly income was highest in the environmental sanitation group (93.5%), followed by the transportation group (79.8%) and the healthcare group (42.6%).

Among the three groups, the healthcare group had the highest proportion of night shift work (52.0%), whereas the environmental sanitation group had the lowest proportion (26.3%). In this study, the proportion of workers with weekly working hours ≤44 h was relatively lower in the healthcare group (49.1%), compared with 55.6% in the environmental sanitation group and 55.5% in the transportation group. Regarding mental health and sleep status, the environmental sanitation group had the lowest prevalence of depressive symptoms (15.3% vs. 27.9%/26.3%), anxiety symptoms (26.8% vs. 44.9%/40.0%), and sleep disturbance (85.1% vs. 94.1%/92.8%). In contrast, the healthcare group had the highest prevalence of depressive symptoms (27.9%), anxiety symptoms (44.9%), and sleep disturbance (94.1%). Detailed overall and group-specific results are shown in Table 1.

**Table 1:** Baseline characteristics of participants across occupational groups.

Variable	Total (*N* = 3,667)	Healthcare (*n* = 1,712)	Environmental sanitation (*n* = 1,022)	Transportation (*n* = 933)	*χ*^2^/*H*	*p*
Sex					1093.752	<0.001
Male	1,344 (36.7)	324 (18.9)	260 (25.4)	760 (81.5)		
Female	2,323 (63.3)	1,388 (81.1)	762 (74.6)	173 (18.5)		
Age, median [P25, P75]	41 [32,49]	34 [29,42]	49 [44,54]	43 [36,49]	931.851	<0.001
Marital status					188.353	<0.001
Married	2,998 (81.8)	1,295 (75.6)	897 (87.8)	806 (86.4)		
Unmarried	426 (11.6)	329 (19.2)	39 (3.8)	58 (6.2)		
Other	243 (6.6)	88 (5.1)	86 (8.4)	69 (7.4)		
Educational level					2482.277	<0.001
High school/ second specialized school or below	1777 (48.5)	87 (5.1)	929 (90.9)	761 (81.6)		
Junior college	645 (17.6)	486 (28.4)	58 (5.7)	101 (10.8)		
Bachelor’s degree or above	1,245 (34.0)	1,139 (66.5)	35 (3.4)	71 (7.6)		
Night shift					196.314	<0.001
No	2023 (55.2)	821 (48.0)	753 (73.7)	449 (48.1)		
Yes	1,644 (44.8)	891 (52.0)	269 (26.3)	484 (51.9)		
Monthly income					870.055	<0.001
<5,000 yuan	2,430 (66.3)	729 (42.6)	956 (93.5)	745 (79.8)		
5,000–6,999 yuan	709 (19.3)	532 (31.1)	34 (3.3)	143 (15.3)		
≥7,000 yuan	528 (14.4)	451 (26.3)	32 (3.1)	45 (4.8)		
Weekly working hours					41.922	<0.001
≤44 h	1927 (52.5)	841 (49.1)	568 (55.6)	518 (55.5)		
45–54 h	1,067 (29.1)	586 (34.2)	257 (25.1)	224 (24.0)		
≥55 h	673 (18.4)	285 (16.6)	197 (19.3)	191 (20.5)		
Depressive symptoms					59.846	<0.001
No	2,788 (76.0)	1,234 (72.1)	866 (84.7)	688 (73.7)		
Yes	879 (24.0)	478 (27.9)	156 (15.3)	245 (26.3)		
Anxiety symptoms					89.510	<0.001
No	2,251 (61.4)	943 (55.1)	748 (73.2)	560 (60.0)		
Yes	1,416 (38.6)	769 (44.9)	274 (26.8)	373 (40.0)		
Sleep disturbance					68.447	<0.001
No	320 (8.7)	101 (5.9)	152 (14.9)	67 (7.2)		
Yes	3,347 (91.3)	1,611 (94.1)	870 (85.1)	866 (92.8)		

Categorical variables are presented as *n* (%), and between-group comparisons were performed using Pearson’s chi-square test. Age was non-normally distributed and was presented as M [P25, P75]; between-group comparisons were performed using the *Kruskal–Wallis H* test. Pairwise comparisons of age were performed using the *Mann–Whitney U* test with Bonferroni correction, and all pairwise differences among the three groups were statistically significant (all *p* < 0.001). Percentages may not sum to 100% because of rounding.

### 3.2 Correlations among study variables

Spearman correlation analysis showed that sleep disturbance was positively correlated with night shift work and the two psychological symptoms of anxiety and depression among the study participants (all *p* < 0.001).

The strongest correlation was observed between anxiety symptoms and depressive symptoms, showing a moderate positive correlation (*ρ* = 0.474). Sleep disturbance was weakly positively correlated with anxiety symptoms (*ρ* = 0.172), depressive symptoms (*ρ* = 0.110), and night shift work (*ρ* = 0.067). In addition, night shift work showed very weak positive correlations with anxiety symptoms (*ρ* = 0.062) and depressive symptoms (*ρ* = 0.063). Except for the relatively evident correlation between anxiety symptoms and depressive symptoms, the correlation coefficients among the other study variables were small, suggesting no strong correlations among these variables (Tables 2–5).

**Table 2:** Spearman’s correlations among study variables (*N* = 3,667).

Variable	Night shift	Anxiety symptoms	Depressive symptoms	Sleep disturbance
Night shift	–			
Anxiety symptoms	0.062^*^	–		
Depressive symptoms	0.063^*^	0.474^*^	–	
Sleep disturbance	0.067^*^	0.172^*^	0.110^*^	–

Values are Spearman’s rho coefficients. Variables were coded as binary indicators.

^*^p < 0.001.

**Table 3:** Fully adjusted main model for factors associated with sleep disturbance estimated using modified Poisson regression with robust variance.

Variable	Comparison	aPR (95% CI)	*p*
Industry	Environmental sanitation workers vs. healthcare workers	0.904 (0.862–0.947)	<0.001
	Transportation workers vs. healthcare workers	0.996 (0.956–1.037)	0.839
Sex	Women vs. men	1.042 (1.012–1.072)	0.006
Marital status	Single vs. married	0.972 (0.940–1.005)	0.092
	Other vs. married	1.031 (0.995–1.068)	0.093
Educational level	College diploma vs. high school/ second specialized school or below	0.998 (0.958–1.039)	0.906
	Bachelor’s degree or above vs. high school/ second specialized school or below	0.994 (0.954–1.036)	0.773
Monthly income	5,000–6,999 yuan vs. <5,000 yuan	0.978 (0.953–1.003)	0.084
	≥7,000 yuan vs. <5,000 yuan	0.972 (0.942–1.002)	0.070
Weekly working hours	45–54 h vs. ≤ 44 h	1.010 (0.987–1.033)	0.415
	≥55 h vs. 44 h	1.001 (0.973–1.030)	0.931
Night shift	Yes vs. no	1.025 (1.003–1.048)	0.028
Age	Per 1-year increase	1.001 (0.999–1.002)	0.433
Anxiety symptoms	Yes vs. no	1.094 (1.073–1.116)	<0.001
Depressive symptoms	Yes vs. no	1.018 (0.999–1.038)	0.068

This main model included anxiety symptoms and depressive symptoms as psychological symptom variables. Occupational stress was examined in separately and mutually adjusted psychological-factor models and is presented in Table 4 and Supplementary Table S4.

**Table 4:** Separately and mutually adjusted associations of psychological factors with sleep disturbance estimated using modified Poisson regression models with robust variance.

Psychological factors	Comparison	Separately adjusted model, aPR (95% CI)	*p*	Mutually adjusted model, aPR (95% CI)	*p*
Anxiety symptoms	Yes vs. No	1.102 (1.083–1.122)	<0.001	1.096 (1.075–1.119)	<0.001
Depressive symptoms	Yes vs. No	1.067 (1.048–1.086)	<0.001	1.021 (1.001–1.043)	0.038

Estimates were obtained from modified Poisson regression models with a log link and robust variance estimator. In the separately adjusted models, each psychological factor was entered into the model individually, with adjustment for industry, age, sex, marital status, educational level, monthly income, weekly working hours, and night shift. In the mutually adjusted model, anxiety symptoms, depressive symptoms, and occupational stress were entered simultaneously, with adjustment for the same covariates. Results for occupational stress are presented in Supplementary Table S3.

**Table 5:** Interaction analyses of occupational group and key exposures in relation to sleep disturbance.

Exposure	Interaction term	aPR (95%CI)	*p*
Anxiety symptoms	Environmental sanitation × anxiety symptoms	1.082 (1.030, 1.136)	0.002
	Transportation × anxiety symptoms	1.002 (0.963, 1.043)	0.908
Depressive symptoms	Environmental sanitation × depressive symptoms	1.049 (0.991, 1.109)	0.098
	Transportation × depressive symptoms	0.998 (0.960, 1.038)	0.935
Night shift	Environmental sanitation × night shift	0.997 (0.938, 1.060)	0.931
	Transportation × night shift	1.034 (0.989, 1.082)	0.142

Healthcare services were used as the reference occupational group. Models were adjusted for age, sex, marital status, educational level, monthly income, weekly working hours, and other corresponding exposures as appropriate.

### 3.3 Factors associated with sleep disturbance

After adjustment for demographic, occupation-related, and psychological factors, sleep disturbance among the three urban operation-supporting occupational groups was significantly associated with occupational group, sex, night shift work, and anxiety symptoms. Compared with healthcare workers, environmental sanitation workers had a significantly lower prevalence of sleep disturbance (aPR = 0.904, 95% CI: 0.862–0.947, *p* < 0.001), whereas no statistically significant difference was observed between transportation workers and healthcare workers (aPR = 0.996, 95% CI: 0.956–1.037, *p* = 0.839). Female workers had a higher prevalence of sleep disturbance than male workers (aPR = 1.042, 95% CI: 1.012–1.072, *p* = 0.006). Compared with non-night shift workers, night shift workers also had a higher prevalence of sleep disturbance (aPR = 1.025, 95% CI: 1.003–1.048, *p* = 0.028).

Anxiety symptoms were significantly associated with a higher prevalence of sleep disturbance in the fully adjusted main model (aPR = 1.094, 95% CI: 1.073–1.116, *p* < 0.001). The association for depressive symptoms was weaker and did not reach statistical significance in this main model (aPR = 1.018, 95% CI: 0.999–1.038, *p* = 0.068). The associations of marital status, educational level, average monthly income, and weekly working hours with sleep disturbance were not statistically significant (all *p* > 0.05).

### 3.4 Separately and mutually adjusted associations of anxiety and depressive symptoms with sleep disturbance

In the separately adjusted models, anxiety symptoms (aPR = 1.102, 95% CI: 1.083–1.122, *p* < 0.001) and depressive symptoms (aPR = 1.067, 95% CI: 1.048–1.086, *p* < 0.001) were both significantly positively associated with sleep disturbance. In the mutually adjusted model including anxiety, depressive symptoms, and occupational stress simultaneously, the effect estimate for anxiety symptoms was partially attenuated but remained significantly associated with sleep disturbance (aPR = 1.096, 95% CI: 1.075–1.119, *p* < 0.001). Depressive symptoms showed a weaker but statistically significant association with sleep disturbance (aPR = 1.021, 95% CI: 1.001–1.043, *p* = 0.038).

### 3.5 Interactions between occupational group and night shift work, anxiety symptoms, and depressive symptoms

Based on modified Poisson regression models with robust variance estimation, multiplicative interactions between occupational group and key exposures were further examined. With the healthcare sector as the reference group, there was a significant multiplicative interaction between the environmental sanitation work and anxiety symptoms (aPR = 1.082, 95% CI: 1.030–1.136, *p* = 0.002), suggesting that the association between anxiety symptoms and sleep disturbance may be stronger among environmental sanitation workers on the prevalence-ratio scale. In contrast, the interaction between the transportation work and anxiety symptoms was not statistically significant (aPR = 1.002, 95% CI: 0.963–1.043, *p* = 0.908), indicating that no obvious difference was observed in the association between anxiety symptoms and sleep disturbance between transportation workers and healthcare workers. In addition, the interaction terms between occupational group and depressive symptoms or night shift work were not statistically significant (all *p* > 0.05), suggesting that there was insufficient evidence to indicate significant differences in the association between depressive symptoms and sleep disturbance across the three occupational groups, and that the association between night shift work and sleep disturbance was generally consistent across the three occupational groups. Interactions involving occupational stress are presented in Supplementary Table S4 and were not statistically significant. To aid interpretation of this interaction, adjusted predicted prevalence of sleep disturbance by occupational group and anxiety symptoms were estimated and are presented in Supplementary Table S9.

## 4 Discussion

### 4.1 Main findings and comparison with previous studies

Based on the 2022 health monitoring data of occupational populations in Chongqing, China, this study compared the prevalence of sleep disturbance across three urban operation-supporting occupational groups, including the healthcare workers (*n* = 1,712), environmental sanitation workers (*n* = 1,022), and transportation workers (*n* = 933). We further examined the associations of night shift work, anxiety symptoms, depressive symptoms, and occupational stress with sleep disturbance.

Significant differences were observed among the three occupational groups in both demographic and work-related characteristics (all *p* < 0.001). Specifically, workers in the healthcare sector had a relatively high proportion of night shift work, those in the environmental sanitation sector had a relatively lower educational level, and the transportation sector was characterized by a higher proportion of male workers. The results showed that the prevalence of sleep disturbance was high in all three occupational groups, at 94.1% in the healthcare sector, 85.1% in the environmental sanitation sector, and 92.8% in the transportation sector, suggesting that urban operation-supporting occupational groups generally face a substantial burden of sleep health problems. This finding may be related to differences in work schedules, labor intensity, psychological stress exposure, and regularity of daily routines across sectors (17, 18).

In the fully adjusted main model, with the healthcare workers as the reference, environmental sanitation workers had a lower prevalence rate of sleep disturbance (*p* < 0.001), whereas the difference between transportation workers and healthcare workers was not statistically significant (*p* = 0.839). This finding suggests that sleep disturbance varied across occupational contexts, although these differences should not be interpreted as independent occupational effects because the three groups differed substantially in sex, education, age, income, work schedules, and other working conditions. Sleep problems were more prominent among healthcare workers, which may be related to long-term high-intensity cognitive work, heavy occupational responsibility, common night shift work, and high occupational psychological stress (2, 11, 12, 31). Previous studies have shown that healthcare workers are exposed to high workloads and irregular work schedules for long periods, and their risk of sleep problems is markedly higher than that of the general working population (31, 32). The prevalence of sleep disturbance among transportation workers was also high, which may be associated with irregular working hours, prolonged driving, and substantial responsibility for transportation safety (33, 34). Notably, although environmental sanitation workers generally had relatively lower socioeconomic status, the prevalence of anxiety and depressive symptoms was relatively low (26.8 and 15.3%, respectively). This finding may be related to the characteristics of their occupational activities. Higher levels of occupational physical activity have been shown to help relieve psychological stress and improve emotional status (35). In addition, higher levels of daytime physical activity may help increase sleep drive and promote circadian rhythm stability (15). Empirical studies in some occupational populations have also shown that regular occupational physical activity is associated with better sleep architecture and psychological status (16). However, the mechanisms underlying this phenomenon cannot be determined from the present cross-sectional data.

In the fully adjusted main model, night shift workers had a higher prevalence of sleep disturbance than non-night shift workers, suggesting that night shift work may be an important occupational factor affecting sleep health, which is consistent with previous studies (9, 36–38). Night shift work may be linked to sleep disturbance through circadian rhythm disruption and the stability of the sleep–wake cycle (10, 39, 40). However, night shift work was measured only as a binary variable in this study. Information on night-shift frequency, duration, rotation pattern, cumulative exposure, and recovery time was not available. Therefore, the observed association should be interpreted cautiously, and future studies should use more detailed measures of shift work exposure. Night shift work is not only common in the occupational groups included in this study, but also prevalent in other urban operation-supporting occupations, such as public security, firefighting, power dispatching, and emergency support (41). Therefore, when developing health management policies for relevant occupational populations, attention should be paid to optimizing night shift systems, reasonably arranging shift rotations, and ensuring sufficient recovery time after night shifts (42, 43).

Regarding psychological factors, anxiety symptoms showed the most stable association with sleep disturbance. In both separately adjusted and mutually adjusted models, anxiety symptoms were significantly associated with sleep disturbance (all *p* < 0.001). Depressive symptoms were also associated with sleep disturbance in the separately adjusted model (*p* < 0.001), but the magnitude of the association was smaller after mutual adjustment. (*p* = 0.038). In the fully adjusted main model, the association for depressive symptoms did not reach statistical significance. These findings suggest that depressive symptoms had a weaker and more model-dependent association with sleep disturbance than anxiety symptoms (21, 44). This finding is consistent with previous findings reported by Alvaro et al. (20).

Previous studies have suggested that anxiety may lead to cognitive hyperarousal, sustained heightened vigilance, and autonomic nervous system activation, thereby affecting sleep initiation and sleep maintenance (20, 45). In addition, anxiety and depressive symptoms are often correlated and may share overlapping symptom dimensions; therefore, the attenuation of the depression estimate after mutual adjustment may reflect symptom overlap rather than the absence of a relationship. The VIF diagnostics did not indicate serious multicollinearity, but the close conceptual relationship between anxiety, depression, and sleep should be considered when interpreting the mutually adjusted models.

The interaction analysis further showed that, with healthcare workers as the reference group, a significant multiplicative interaction was observed between the environmental sanitation work and anxiety symptoms after full adjustment for age, sex, educational level, and other corresponding exposure factors (aPR = 1.082, 95% CI: 1.030–1.136, *p* = 0.002). This finding suggested that the association between anxiety symptoms and sleep disturbance may be stronger among environmental sanitation workers on the prevalence-ratio scale. The adjusted predicted prevalence estimates in Supplementary Table S9 supported this interpretation in absolute terms, although they were not treated as formal additive interaction indices. This interaction also remained significant in the sensitivity analysis using a stricter sleep disturbance definition, supporting the robustness of this signal. The sensitivity analysis using a stricter definition of sleep disturbance yielded a lower prevalence estimate but generally consistent findings regarding the interaction between anxiety symptoms and environmental sanitation work, suggesting that these findings were not solely explained by the primary low-threshold definition. Nevertheless, the observed interaction did not establish that environmental sanitation work amplified the effect of anxiety symptoms on sleep disturbance. One possible explanation was that, because environmental sanitation workers had a lower baseline prevalence of sleep disturbance and lower overall levels of anxiety and depressive symptoms, the relative difference associated with anxiety symptoms may appear more pronounced in this group. However, this explanation is speculative and was not directly tested. Further longitudinal studies with more detailed occupational exposure assessment are needed to confirm this occupational group-specific pattern.

### 4.2 Strengths and limitations

This study has several practical implications. Previous studies have mostly focused on single occupational populations. By focusing on urban operation-supporting occupational groups, this study compared differences in the distribution of sleep disturbance across occupations and explored related influencing factors, which may help enrich the evidence on sleep health among occupational populations.

This study also has several limitations. First, the cross-sectional design precluded assessment of temporality or causality; therefore, the findings should be interpreted as associations rather than causal effects. Second, all data in this study were collected through self-reported questionnaires, which may have been affected by recall bias, reporting bias, and social desirability bias. Third, sleep disturbance was assessed using only three sleep-related items, and the primary cut-off of ≥1 was sensitive and may have contributed to the high prevalence. Although a stricter cut-off of ≥2 was used in sensitivity analyses and yielded generally consistent findings, outcome misclassification remains possible. Fourth, night shift work was measured as a binary variable, and information on night-shift frequency, duration, rotation pattern, cumulative exposure, and recovery time was not available. This may have led to exposure misclassification and limited our ability to examine dose–response associations. Fifth, although this study adjusted for multiple demographic and occupation-related factors, some potential confounders may not have been fully controlled, such as chronic disease status, physical activity, smoking, and alcohol consumption among workers. Several unmeasured factors may have affected the direction or magnitude of the reported associations. Chronic disease status, medication use, smoking, and alcohol consumption may be related to both psychological symptoms and sleep disturbance (46, 47), and their absence from the models may have resulted in residual confounding, potentially overestimating the associations between psychological symptoms and sleep disturbance. Unmeasured differences in physical activity or general health status may also partly explain the lower prevalence of sleep disturbance among environmental sanitation workers (48). Because these factors were not collected, their exact influence on the direction and magnitude of the observed associations could not be quantified. Finally, the study participants were derived from a specific monitoring project in Chongqing, China, and the sample had certain limitations in terms of region and occupational scope. Therefore, caution is needed when generalizing the findings to other regions or occupational populations.

Although the interaction term and predicted prevalence estimates suggested that the association between anxiety symptoms and sleep disturbance may differ by occupational group, the present data does not establish the underlying mechanism. Further longitudinal studies with more detailed occupational exposure assessment are needed to confirm this finding.

## 5 Conclusion

This study found that the prevalence of sleep disturbance was relatively high among all three urban operation-supporting occupational groups, with certain differences across occupational groups. Night shift work and anxiety symptoms were associated with sleep disturbance, with anxiety symptoms showing the most stable association. Depressive symptoms showed a weaker and model-dependent association. The significant interaction between environmental sanitation work and anxiety symptoms suggests that the anxiety–sleep disturbance association may vary by occupational context. These findings suggest that sleep health and mental health management should be incorporated into occupational health services for urban operation-supporting occupational groups, with differentiated strategies tailored to specific occupational groups.

## Data Availability

The raw data supporting the conclusions of this article will be made available by the authors, without undue reservation.
