# Supplementary material for: Sleep disturbance and its associations with night shift work, anxiety symptoms, and depressive symptoms among urban operation-supporting workers: a cross-sectional study
Source: Front Public Health. 2026 Sep 29;14:1935598. doi: 10.3389/fpubh.2026.1935598 (PMC13658507; doi:10.3389/fpubh.2026.1935598)
Supplement: Supplementary file 1 [file Supplementary_file_1.pdf]

Supplementary Material

1 Supplementary Data

The main text of this study focuses on the primary associated factors and occupational heterogeneity findings. The full analysis of occupational stress as a psychological factor in relation to sleep disturbance is reported as a supplementary analysis to avoid excessive length in the main text.

This supplementary material provides additional analyses to complement the main findings, including the associations between occupational stress and sleep disturbance in separately adjusted and mutually adjusted models, the interaction between occupational group and occupational stress, and the multicollinearity diagnostics of relevant variables in the multivariable regression models.

Occupational stress (OS): Occupational stress among primary and secondary school teachers was assessed using the *Core Occupational Stress Scale* (COSS). The scale was developed by the National Institute for Occupational Health and Poison Control, Chinese Center for Disease Control and Prevention, and has been shown to have good reliability and validity for assessing occupational stress in Chinese populations. The scale contains 17 items and includes four dimensions: social support, organization and reward, demand and effort and autonomy. It is scored on a 1–5 scale and includes items on social support and autonomy (including reverse-scored items). A total score ≥ 50 was considered indicative of OS, and higher total scores indicate higher levels of occupational stress. The Cronbach's alpha of the scale was 0.773.

The distribution of occupational stress differed significantly among the three occupational groups. The prevalence of occupational stress was the highest in the transportation sector (35.2%), followed by the environmental sanitation sector (29.0%) and the healthcare sector (23.0%), with a statistically significant between-group difference (χ^2 = 45.913, $P < 0.001$).

Correlation analysis showed that occupational stress was positively correlated with anxiety and depression symptoms (ρ = 0.257 and 0.297, respectively; both $P < 0.001$), suggesting certain correlations between occupational stress and psychological symptoms. However, the correlation between occupational stress and sleep disturbance was weak and not statistically significant (ρ = 0.021).

In the separately adjusted models, the association between occupational stress and sleep disturbance was not statistically significant (aPR = 1.018, 95% CI: 0.996 – 1.041, $P = 0.113$).In the mutually adjusted model including anxiety symptoms, depressive symptoms, and occupational stress simultaneously, occupational stress showed no significant independent association with sleep disturbance (aPR = 0.984, 95% CI: 0.962 – 1.008, $P = 0.205$).

Further interaction analysis showed that the interaction terms between occupational group and occupational stress were not statistically significant. The aPR for environmental sanitation × occupational stress was 0.963 (95% CI: 0.905 – 1.026, $P = 0.241$), and the aPR for transportation × occupational stress was 1.024 (95% CI: 0.981 – 1.070, $P = 0.278$). These results suggest that, although the distribution of occupational stress differed across occupational groups and occupational

stress was correlated with psychological symptoms such as anxiety and depression, no significant independent association between occupational stress and sleep disturbance was observed after full adjustment for relevant confounders.

2 Supplementary Tables

Supplementary Table S1. Baseline characteristics of participants across occupational groups.

Variable	Total (N=3667)	Healthcare （n=1712）	Environmental sanitation （n=1022）	Transportation （n=933）	χ2	*P*
**Occupational stress**					45.913	＜0.001
No	2650 (72.3)	1319 (77.0)	726 (71.0)	605 (64.8)		
Yes	1017 (27.7)	393 (23.0)	296 (29.0)	328 (35.2)		

Supplementary Table S2. Spearman’s correlations among study variables （N=3667）

Variable	Night shift	Anxiety symptoms	Depressive symptoms	Occupational stress	Sleep disturbance
**Night shift**	-				
**Anxiety symptoms**	0.062**	-			
**Depressive symptoms**	0.063**	0.474**	-		
**Occupational stress**	-0.028	0.257**	0.297**	-	

**Sleep disturbance**	0.067**	0.172**	0.110**	0.021	-

Note: Values are Spearman's rho coefficients. Variables were coded as binary indicators.

*$P < 0.05$ **$P < 0.001$

Supplementary Table S3. Associations of anxiety symptoms, depressive symptoms, and occupational stress with sleep disturbance estimated using modified Poisson regression models with robust variance

Psychological factors	Comparison	Separately adjusted model, aPR (95% CI)	*P*	Mutually adjusted model, aPR (95% CI)	*P*
**Anxiety symptoms**	Yes vs No	1.102 (1.083 – 1.122)	<0.001	1.096 (1.075 – 1.119)	<0.001
**Depressive symptoms**	Yes vs No	1.067 (1.048 – 1.086)	<0.001	1.021 (1.001 – 1.043)	0.038
**Occupational stress**	Yes vs No	1.018 (0.996 – 1.041)	0.113	0.984 (0.962 – 1.008)	0.205

Supplementary Table S4. Interaction analyses of occupational group and key exposures in relation to sleep disturbance

Exposure	Interaction term	aPR (95% CI)	*P*
**Anxiety symptoms**	Environmental sanitation × anxiety symptoms	1.082 (1.030 – 1.136)	0.002
	Transportation × anxiety symptoms	1.002 (0.963 – 1.043)	0.908
**Depressive symptoms**	Environmental sanitation × depressive symptoms	1.049 (0.991 – 1.109)	0.098
	Transportation × depressive symptoms	0.998 (0.960 – 1.038)	0.935
**Night shift**	Environmental sanitation × night shift	0.997 (0.938 – 1.060)	0.931
	Transportation × night shift	1.034 (0.989 – 1.082)	0.142
**Occupational stress**	Environmental sanitation × occupational stress	0.963 (0.905 – 1.026)	0.241
	Transportation × occupational stress	1.024 (0.981 – 1.070)	0.278

Note: Healthcare workers were used as the reference occupational group. The ratio of aPRs represents the multiplicative interaction term estimated using modified Poisson regression with robust variance. Models were adjusted for age, sex, marital status, educational level, monthly income, weekly working hours, and other corresponding exposures as appropriate.

Supplementary Table S5. Multicollinearity diagnostics for independent variables

Variable	Tolerance	VIF
Age	0.586	1.707
Sex	0.877	1.141
Marital status	0.958	1.044
Educational level	0.509	1.965
Monthly income	0.726	1.378
Weekly working hours	0.899	1.113
Night shift	0.862	1.160
Depressive symptoms	0.734	1.363
Anxiety symptoms	0.740	1.351
Occupational stress	0.865	1.157

Note: VIF, variance inflation factor. All variables showed VIF values below 2.0, suggesting no evidence of serious multicollinearity among the independent variables.

Supplementary Table S6. Prevalence of sleep disturbance according to primary and stricter definitions by occupational group

**Occupational group**	**N**	**Primary definition: cut-off ⩾1, n (%)**	**Stricter definition: cut-off ⩾2, n (%)**
Healthcare workers	1,712	1,611 (94.1)	1,424(83.2)
Environmental sanitation workers	1,022	870 (85.1)	688 (67.3)

Transportation workers	933	866 (92.8)	740 (79.3)
Overall	3,667	3,347 (91.3)	2,852 (77.8)
P value		<0.001	<0.001

Note: The primary definition of sleep disturbance was a score of ⩾1, and the stricter definition used in sensitivity analyses was a cut-off of ⩾2. *P* values were obtained from chi-square tests.

Supplementary Table S7. Sensitivity analysis using the stricter sleep disturbance definition of the score of ⩾2

**Variable**	**Comparison**	**aPR (95% CI)**	***P***
Industry	Environmental sanitation workers vs healthcare workers	0.811 (0.754 – 0.871)	<0.001
	Transportation workers vs healthcare workers	0.959 (0.899 – 1.023)	0.208
Sex	Women vs men	1.070 (1.022 – 1.120)	0.004
Night shift	Yes vs no	1.037 (0.999 – 1.075)	0.054
Anxiety symptoms	Yes vs no	1.287 (1.244 – 1.332)	<0.001
Depressive symptoms	Yes vs no	1.065 (1.032 – 1.100)	<0.001

Note: Estimates were obtained from modified Poisson regression models with a log link and robust variance estimator. The model was adjusted for occupational group, age, sex, marital status, educational level, monthly income, weekly working hours, night shift, anxiety symptoms, and depressive symptoms.

Supplementary Table S8. Sensitivity analysis for the interaction between occupational group and anxiety symptoms

Interaction term	aPR (95% CI)	*P*
Environmental sanitation × anxiety symptoms	1.133 (1.039 – 1.235)	0.005
Transportation × anxiety symptoms	1.047 (0.972 – 1.127)	0.226

Note: Healthcare workers were used as the reference occupational group. Estimates were obtained from modified Poisson regression models with a log link and robust variance estimator. The model was adjusted for age, sex, marital status, educational level, monthly income, weekly working hours, night shift, depressive symptoms, occupational group, anxiety symptoms, and the corresponding interaction terms.

Supplementary Table S9. Adjusted predicted prevalence of sleep disturbance by occupational group and anxiety status

Occupational group	Anxiety symptoms	Adjusted predicted prevalence (95% CI)
Healthcare workers	No	0.91 (0.88 – 0.94)
Healthcare workers	Yes	0.98 (0.95 – 1.00)
Environmental sanitation workers	No	0.80 (0.76 – 0.84)

Environmental sanitation workers	Yes	0.93 (0.89 – 0.97)
Transportation workers	No	0.90 (0.87 – 0.94)
Transportation workers	Yes	0.97 (0.94 – 1.00)

Note: Adjusted predicted prevalences were estimated from modified Poisson regression models with a log link and robust variance estimator using the primary sleep disturbance definition. The model included occupational group, anxiety symptoms, the occupational group × anxiety interaction term, age, sex, marital status, educational level, monthly income, weekly working hours, night shift, and depressive symptoms. These predicted prevalences were used to aid interpretation of absolute differences and were not interpreted as formal additive interaction indices.
